# Supplementary material for: Food insecurity in a Brazilian transgender sample during the COVID-19 pandemic
Source: PLoS One. 2023 May 10;18(5):e0284257. doi: 10.1371/journal.pone.0284257 (PMC10171619; doi:10.1371/journal.pone.0284257)
Supplement: Attachment — Submitted filename: Response to reviewers.docx [file pone.0284257.s001.docx]

**Response to reviewers**

Dear Editor,

We appreciate the careful reading of our manuscript. We have prepared a revised version of the paper that considers all the comments the associate editor and reviewers made.

We changed the discussion to include more information on gender and food insecurity, considering the data at national levels, including the perspective of social determinants of health. In addition, we made changes in writing some points of method and results to improve the comprehension of the text. In the end, we answered point-by-point the reviewer's comments.

We look forward to receiving your decision.

Sincerely,

Sávio Gomes

(on behalf of the authors)

Reviewer's Responses to Questions

**Reviewer #1:**

1) During your introduction I read about the relationship between gender and food insecurity, nevertheless, this point was not fully addressed during your discussion and how interacts with other related factors. It is important to include how your results compare to the national ones (as you used the same scale used in the national surveys), from this information it is easier to understand the results with this population.

*Answer: Done. We included more information on gender and food insecurity, considering the data at the national level. See the third paragraph of the discussion.*

2) In general terms, I considered that regarding the information that you had the opportunity to collect you are not fully explaining some interactions between food insecurity and social determinants of health. For example, it is interesting that 55% of your population is above the average schooling level while 68.8% experienced some degree of food insecurity did you check on your models how these two factors interact? Are they related? I encouragingly suggest going deeper inside those SDH that is related to food insecurity then you can explain more about how it is for these group the experience food (in)security.

*Answer: Done. See paragraph 5 of the discussion.*

3) It is a fact that during your study, there was not a direct correlation between prejudice and food insecurity. Nevertheless, it must be taken into account that food insecurity is a multifactorial problem, and those related to social exclusion are considered part of the causes. I suggest including as a recommendation for further studies to use methodologies that allow showing how prejudice impacts the factors mentioned in line 244 and, as a result, the level of food insecurity in this group.

*Answer: Done. We included these issues in the conclusion.*

**Reviewer #2:**

4) In several analyses, such as in Table 1, participants are categorized as having food security or having food insecurity. It was not entirely clear how these were defined.

*Answer: Done. We specified this information in the second paragraph of the Instruments and variables, methods section.*

5) Figure 2 shows that around 30% of the individuals in the food security group reported mild food insecurity and around a similar percentage in the food insecurity group reported mild food insecurity – the latter group also includes individuals with moderate or extreme food insecurity.

*Answer: In figure 2, the first column is about just people who experienced food security, and the second column is about just people who experienced food insecurity, so in the food insecurity group, it is true that around 30% reported mild food insecurity. We perceived that the color was incorrect in the first column and changed it.*

6) In the first half of the manuscript, it indicates that “internalized prejudice” was assessed. This normally refers to the attitudes or emotional states of an individual toward themselves which is a reflection of negative attitudes found in the person’s family, community, or country. An example would be a trans person who believes trans people are bad or inferior or has a low self-esteem as a result of being trans because that is what the person has been told by their society. However, the items in that measure appear to assess the experience of interpersonal discrimination – e.g., from employers, police, or others, rather than the internal state of the individuals.

The second half of the manuscript generally refers to this variable as “perceived prejudice” which seems the more appropriate term. It would be preferable to use just one term for this variable consistently throughout the manuscript.
*Answer: That true. We adopted perceived prejudice in the entire manuscript.*

7) Table 1 shows a marginal association between prejudice and food insecurity (p = .08) and the distributions seem quite different. Given the importance of this variable, were additional analyses conducted, such as assessing if prejudice was associated with extreme food insecurity rather than just food insecurity overall?

*Answer: We agree, and we tried to conduct analyses to test the association between prejudice and extreme food insecurity, but because of the number of categories in both variables (perceived prejudice and food insecurity), the number of cases in the table is insufficient to perform rigorous statistical analysis. We included in the conclusion the suggestion that it is necessary to conduct research with a bigger sample to test the differences between categories.*

8) A minor point, but the Table 1 header does not appear to actually describe the data presented in that table.

*Answer: Done.*

In addition: All files are available from the OSF database. DOI: 10.17605/OSF.IO/YHNEM.
